# Extra-Limites

**Published:** 1825-01-01

**Authors:** 


### XIII.

### EXTRA LIMITES.*

### I.

### AUTO-ADVERTISING, AND MEDICAL DEGREES.

*To the Editors of the Medico-Chirurgical Review.*

GENTLEMEN,

From the tenor of your Journal, I conceive it is your inclination, as it, unquestionably, is your duty, to pay attention to the *dignity*, and consequently the *interests*, as well as to the *Science* of Medicine and its professors. I need not inform you, Gentlemen, that those who practise the healing art, are, unhappily, obnoxious to much illiberality from abroad, and some temptation from within. The world is censorious enough, even where no cause is given for censure. There is a general prepossession against Physic, as well as against Law—and few, I believe, have recourse to one or the other, but from necessity. For this, and for various reasons, medical men ought to be exceedingly cautious in resorting to any measure which may tend to lower the dignity of the science, however advantageous it may be to themselves individually. It is the object of this paper to touch on an evil, which has increased at a prodigious rate, of late years, and which is now becoming so much the subject of public remark, as to threaten a general odium on the pro-

* N.B. As no explanation of the nature and objects of the EXTRA-LIMITES department of this Journal has been given in this New Series, it may be proper to say a few words on the subject.

When this Work changed its form, from a Miscellaneous Repository to that of a *Review* exclusively, it was hinted that gentlemen would occasionally wish to obtain extensive circulation for defences against criticisms, or even original discoveries, through the medium of the MEDICO-CHIRURGICAL REVIEW. It was obvious that such papers must be beyond the regular limits of the Work, and consequently gratis to the public; otherwise the plan would be broken up, and the Journal cease to be a Review. With the design of accommodating individuals, without detriment to the subscribers or the conductors of the Journal, the EXTRA-LIMITES department has been established, in which every man may *claim* a place for his observations, provided they are couched in temperate language, and not containing personal, offensive, or libellous matter. Over *these*, the Editor must necessarily exercise a control; but in every thing else, the EXTRA LIMITES is neutral ground, where the criticisms of this, as well as of other Journals, may be freely discussed and combated.

It is only necessary, in conclusion, to observe, that the expense of paper and type falls on the authors of the papers.

fession: I mean the now prevailing practice of medical men *advertising themselves*. This custom is, at present, so universal, that it is no easy matter for the public to draw the line of discrimination between the REGULAR and the QUACK. Indeed, as far as the expedient of advertising is concerned, I defy any man to draw a satisfactory distinction between the one and the other! I do not mean to say that a Physician's or a Surgeon's name should *never* appear in an advertisement; because the thing is sometimes unavoidable; but I will venture to draw a line of demarcation between proper and improper—that is, between honorable and venal *modes* of advertising in the public prints.

*First.* MEDICAL ELECTIONS. When a candidate for an hospital or dispensary commences his canvass, is it proper to address the governors and patrons of such institutions, through the medium of a newspaper? The propriety of this practice is very questionable. The Royal College of Physicians discountenances this practice among its *Fellows*—and it will be allowed by all, that the College studies, with no small degree of intensity, whatever conduces, or is supposed to conduce, to the respectability and interests of its own body. I, therefore, am inclined to think the College right in this respect, but wrong in not extending the same regulation to the Licentiates. Let us reason for one moment on this point: The candidate for a public institution canvasses the governors privately and individually—and justifiably lays before them such documents as bear on his professional and moral character. This is enough. All addresses to the Public at large are unnecessary, as far as the immediate object of the candidate is concerned, and certainly appear to me to approach the verge of AUTO-ADVERTISEMENT. If this term, however, applies not to the simple avowal of a candidate's intention to canvass for a public institution, yet I think it will fairly apply, when the said candidate couples with such avowal a recitation of his own merits—of the experience he has had—and of the high opinions which have been formed of him—and all this through the medium of an advertisement in a public newspaper?—this, I maintain, is *Auto-advertisement.** In short, Gentlemen, it is now notorious that, when a vacancy occurs in a public institution, a whole swarm of candidates start forth—not for the purpose of canvassing the electors—but solely with the view of advertising themselves. Is this consistent with the dignity of the medical profession?

*Secondly. Medical Lectures.* I have said that it is sometimes unavoidable that the names of physicians and surgeons should appear in

* M. Biot, of the Institute, sometime ago drew a good distinction between the philosophical and charlatanic physician. Speaking of the *latter*, he observes—"Le Charlatan au contraire, a besoin de dehors qui frappent le peuple et qui previennent l' examen. Loin de s'addresser à des juges éclairées, il les recuse, il les taxe d'une severité exagerée, souvent même d'envie et d'injustice; c'est á la multitude qu'il en appelle. *Les feuilles publiques sont la théâtre éphemére où il établit sa renommée.*"

public advertisements. It is proper and necessary that Lecturers—actual Lecturers—should announce their courses through the medium of the public prints, at proper times and seasons. But when men who are notorious for not having a single pupil, occupy with their advertisements the *sinister* corner of every fashionable paper, and that for weeks and months—have we not a right to put down such practice under the head of *Auto-advertisement ?* Even the regular lecturer, with a fair class of auditors, should be economical of his reputation, as well as of his purse, in resorting *too often* to this measure.

*Thirdly. Medical Books.* This mode of *Auto-advertisement* is the oldest, most extensive, and best understood of any. It may, indeed, be considered as the parent stock, from which all the others have sprung. I do not, of course, allude to the myriads of publications daily issuing from those who are out of the pale of the profession. These are instantly recognized, and readily appreciated by the republic of medical science. They impose on the *people* and probably will for ever continue to do so. I here allude to the practice of *making* a book upon some particular disease, or class of diseases—totally regardless of its reception among the real judges—and with the sole view of having something to *make* an *advertisement* of, and keep the name of the author constantly before the public, in the newspapers. I would not be considered, Gentlemen, as censuring any thing but *abuses.* If a book be published, it must be advertised, and in that way the names of all that grace our Science have come, and daily come, before our eyes, in the public prints. This is unavoidable. But when, on the advertisement of a book, ten times more money is spent than the sale of it can possibly cover—then, I say, and confidently affirm, that the principle and spirit of *Auto-advertising* are in full operation.

*Fourthly.* There is one subject which I purposely left unnoticed, when speaking of Medical Elections, in order that I might give it a fuller consideration under a separate head. It is stated, in justification of the appeal of the *Licentiate* to the public, through the medium of the newspapers, in cases of election, that he has no other chance left, when contesting the point with the *Fellow* of the College, since the *latter* is sure to have the whole influence of the College thrown into the scale against him. This is complained of, as a great evil—and if it exist, which I am not prepared to deny, it must be confessed that it is a very great one. So mighty and preponderating, indeed, are the disadvantages under which the *Licentiate* labours—so clearly and generally is it now known that he has *no* chance of appointment to a public hospital—and comparatively little hope of success in private practice in this metropolis—that I conceive the evil must cure itself, in the course of the next twenty years.* I am of opinion that the evil will find remedy in

* From the document which accompanies this paper, you can vouch, Gentlemen, that I now belong to neither class above alluded to, and consequently that my sentiments are, at least, free from bias to either side.

a way very different from that contemplated by the present race of Licentiates. It will never be cured, nor is it desirable that it should be cured, by forcing concessions from the College. It will be remedied by a general knowledge of the advantages attendant on a fellowship—and the disadvantages under which the Licentiate labours. Henceforth, no man who can possibly avoid it, will educate his son with the view of practising as a physician in this metropolis, but through the channel of a fellowship. It would be madness to do otherwise. I venture, therefore, to predict that, from this time forward, the proportion of Fellows will annually increase, and that of Licentiates diminish. A time must consequently come, and that at no distant date, when the fellowship will be so extended, as to remove any thing like competition or clashing, between the two classes of physicians.

But it will be said, and with reason, that, in the mean time, many men of great talents, integrity, and acquirements, will unmeritedly suffer. Not a doubt of it. But this will always be the case, and it can be no good reason why improper, temporary, or unavailing remedies should be had recourse to.

When it is considered that the influx of men into the medical profession is far out of proportion to the demand—that this crowded state is productive of great evil to the actual practitioners, forcing many of them on ways and means not calculated to heighten the dignity of the faculty—it will surely be granted, that any check to such influx, founded on an extension of general and scientific education, is both salutary and desirable. On this account, I maintain that it is far better the necessary academical education of the physician should rise to that of the Fellow, than remain, as it now does, without any fixed standard, as in the case of the Licentiate.

I have said that, hereafter, the list of Fellows will increase much more in proportion than that of the Licentiates. This must be the case, when the immense advantages of the one, and disadvantages of the other, come to be properly appreciated by the public at large—and especially by those members of the profession who design their sons for the station of physician, with the chance of practising in the metropolis. Now, let us inquire what are the sacrifices or expences necessary to secure a fellowship, instead of a licence. It is nothing more than giving a youth three years' University education, as a preparatory for his medical studies, instead of the same number of years spent in the private acquisition of classical and general knowledge. Let me explain. Suppose a physician, surgeon, or general practitioner, residing in London, Dublin, or Edinburgh, designs his son for a physician. At the age of 18 or 19, after a fair scholastic education, he sends him to Oxford, Cambridge, or Trinity College, Dublin, for three years—at an expence within two hundred pounds per annum. At the expiration of this period, he takes a Batchelor's degree, and no more actual residence at the University is required. He now returns to his paternal roof, and applies himself for four years to the study of medicine in all its branches, either in London, Dublin, or Edinburgh, where the parent may happen to reside. At the end of these four years, he is in a situation to commence practice (and it is early enough at the age of 25 or 26) as an inceptor candidate, with the certain prospect of becoming a fellow of the College at the age of thirty. Now, Gentlemen, I would ask you, and the profession at large, whether the above course is not better calculated to make a good and a respectable physician, than the one now generally pursued by the Licentiate—and yet at an expense very little exceeding that which attends the diploma of the latter. Is not a good scholastic education necessary?—Is not three years' University education highly desirable?—Is not four years study of all the branches of medical science, *in London*, as good (to say no more) as the same, or half that period spent in Edinburgh or Glasgow?—no man of unbiassed judgment will answer in the negative. And I will venture to affirm that no medical man, who designs his son for a physician, and who has the welfare of his son at heart, will fail, in future, to push his classical, academical, and medical education through the channel which I have pointed out, in order to secure him against the mortifying degradation which he must expect as a *Licentiate* in London.

I may here suggest, that the College of Physicians should, in addition to the University course, require proofs of four years' regular study in London, Dublin, Edinburgh, Glasgow, or Paris. If they did this, they would for ever stop the mouths of those who rail at degrees taken where no efficient school of medicine exists.

*Lastly.* It is a melancholy truth, that there is no good in this world, without some attendant evil. While the abovementioned course of studies will draw physicians from a respectable class of society—render them learned and well-grounded in the elements of their science—raise them in public estimation—and prove a check on all deviations from strictly professional conduct—it must be conceded that it will bear hard on many a genius whose "res angusta domi" compels him to seek an humbler path to the *summi honores* of medicine! But this must ever be the case, with the wisest and best of human laws and human institutions. The GOOD attendant on raising the general body of physicians in respectability, far counterbalances the EVIL of repressing a few choice spirits. But, even in this view of the subject, there is a consolation which rests on the history of all ages and countries. TALENT, of a certain order, will surmount all difficulties—and more than surmount them—it will shine the more conspicuous in proportion to the magnitude of the obstacles which have come in its way.

Withdrawn from the cares and anxieties of professional avocations, I am still an attentive observer of what passes in the medical world, and I can conscientiously declare, a sincere well-wisher of the profession to which, for nearly 40 years, I belonged.

I am, Gentlemen,

Your most obedient servant,

PHILO-MEDICUS.

*Hampstead, Dec.* 1, 1824.

### II.

### MR. HICKES ON PREGNANCY AND ASCITES.

*To the Editor of the Medico-Chirurgical Review.*

SIR,—Your statement of Mr. Langstaff's inability to procure advice, in a case of *ascites* in pregnancy, co-operating with solicitude for preservation of human life, even in an embryo state, counteracts my habitual reluctance to obtrude any novel mode of treatment on public attention.

Two cases of peculiar delicacy, incidentally required my exertion in capacity of accoucheur, although, for several years, the extent of my professional avocations has precluded my retention of that branch of practice.

About two years since a case of *ascites*, concurrent with pregnancy, was submitted to my care, subsequently to denial of pregnancy by one of the most eminent in the profession. Reluctant, through subduction of the *liquor amnii*, to destroy the fœtus, I ventured, on my own responsibility, even unencouraged by precedent, consultation, or recorded authority, to originate operation for *ascites*, on a pregnant patient. I was, in the first instance, influenced by consideration of the ordinary transmission of blood, through the *placenta* to the *uterus*, being unavoidably checked, on the exterior layer of its muscles, with their integuments, being brought into contact with the *parietes* of the *uterus*, through loss of the *liquor amnii*. In the next place, the *diagnosis* of impregnation being, to my perception, indisputable, I was solicitous to relieve tension of the abdomen, by exhausting the redundant lymph in the abdominal absorbents. Through such operation, I therefore anticipated a triple effect;—immediate alleviation of the patient's extreme suffering—facilitation of approaching parturition—with preservation of life both to parent and offspring. It was an experiment—if bold, by no means adventurous, and it terminated in the gratifying result of complete eventual success.

In corroboration of the preceding statement, I feel it due, not less to professional character, than to excitement of similar operation in parallel cases, to refer the reader, desirous of ulterior proof, to a letter from my patient, dated January 17th, 1823, in consideration of its author's rank, with greater propriety lodged in your possession for inspection of the scientific, than subjected to indiscriminate publicity. Similar treatment of a subsequent case has likewise occurred in my practice.

A lady of Cornwall, returning from Italy, became decrepid during the second month succeeding to cessation of menstruation. Internal abscesses were developed, through emission of purulent matter from the anus. The consequent debility was excessive. Indeed, the general symptoms presented to the surgeon of the packet indication of approaching termination of life. In such deplorable state, during the fourth month of unsuspected pregnancy, she placed herself under my direction. Indication of *ascites* in an early stage was decisive—the symptomatic affections were, frequent recurrence of spasm—oppressive respiration in an horizontal posture—with the ordinarily attendant complaints. In the lapse of three or four weeks, the fœtus, acquiring competent dimension, detected its head to the usual test, application of the hand. In contempt of such incontrovertible prognosis, the impression of improbability of conception supervening, subsequently to eight or nine years of infecundity, could not be eradicated from the patient's apprehension. It should be stated that, from the period of impregnation, conjugal intercourse fortuitously terminated. Necessity of *early* operation, as in the preceding case, was by no means indicated. Repletion of the abdominal absorbents advanced with inferior progress. Tension on the lungs was felt with less degree of inconvenience. The symptoms in general, certainly unambiguous, were yet unattended with equal oppressiveness. The object of *paracentesis* being principally to facilitate parturition, although, in part of course, to remove specific disease, the operation was postponed to a week or ten days previous to commencement of labour pains. On delivery, the child appeared full-grown, perfect, and of the male sex; but through some undiscoverable cause of excitement, probably adventitious, and unconnected with influence of *ascites*, the umbilical cord, having surrounded the neck, had produced the usual result, strangulation. Operation of some foreign incidental cause was equally suggested by an additional indication—adhesion of the *placenta* to the *parietes* of the *uterus*. The flooding becoming excessive, constrained extraction, through introduction of the hand, became indispensible as an exclusive expedient. Notwithstanding the minutest caution observed in removal of the adherent portions of *placenta*, the consequent inflammation of the uterus required exhibition of refrigerant and antiphlogistic treatment. It was preceded by bleeding, as well through venesection, as through local application of leeches. In the course of three or four months, asthenic symptoms disappearing, the cure of this delicate case attained its consummation.

In a patient presenting such remarkably asthenic symptoms, had not parturition been preceded by *paracentesis*, its pains might have been rendered too excruciating, its duration too prolonged, to be encountered without hazard of life.

One observation remains, equally applicable to both cases: In neither, has the most distant indication of recurrent *ascites* been detected. In the first instance, two years, in the second, several months have elapsed without alarm; consequently, *paracentesis* was not merely warranted, by production of instantaneous relief from pain, but was absolutely necessary for attainment of radical recovery.—Delay of the operation to any given period, subsequent to parturition, while it inevitably would have augmented the patient's suffering pending labour, would probably have permitted the morbid torpor of the abdominal absorbents to extend beyond possibility of re-invigoration. In such event, irrecoverable loss of tone necessarily supervening—the patient's life could not fail to have fallen a sacrifice to timid procrastination.

I have the honour to be, Sir,

Your obedient servant,

Bath, November 27, 1824. CHARLES HICKES.

*Note by the Superintending Editor.*

The letter alluded to is in the possession of Dr. Johnson, and no doubt of its authenticity can be entertained. Mr. Hickes will observe that, in the second Number of this series, *several cases* of the kind in question have been brought forward, and where paracentesis was successfully performed. Mr. Langstaff therefore might have found precedents had he searched for them. Another precedent can now be pointed out, which was recorded 80 years ago, and may be seen in the 5th volume of the Medical Essays and Observations, page 642 *et seq.* The case is related by Mr. Laurie, surgeon in Selkirk, who called in the celebrated Dr. Plummer in consultation. The female was 36 years of age, and was three times tapped during pregnancy. She brought forth a living child, and entirely recovered from the ascites. This point of obstetric practice may now be considered as settled, the precedents being quite numerous enough to form a basis for the general principle.

P.S. Since writing the above, a paper has appeared in the Medical and Physical Journal for December, from Sir L. Maclean, pointing out another case of ascites and pregnancy, which was published many years ago in that gentleman's invaluable work on hydrothorax. As this work is by no means so universally read as it ought to be, and as it is probable that, comparatively, few of the readers of this Journal have it in their possession, an abstract of the case may not be unacceptable here, in order to render our knowledge of the subject as extensive as possible.

*June 13th*, 1791, Mrs. C. aged 34, was found by Sir L. with confirmed ascites, unaccompanied by anasarca. She was emaciated, but not proportionally debilitated. The origin of the dropsical affection was dated two years back, since which the catamenia had been irregular. Various remedies had been used, and she had been tapped three times—the last only four days prior to the above date. Sir L—— ascribed the dropsy to disease of the right ovarium. On his next visit (9th August) it was suspected (by the patient) that pregnancy also existed. She was, however, tapped at this time, and towards the middle of August pregnancy was proved by the occurrence of quickening. On the 25th September, paracentesis was again necessary, taking care to avoid injuring the uterus. No untoward circumstance occurred during or subsequent to the operation. By November the 9th the lady was labouring under great distress from distention of the abdominal integuments. The operation again performed. December 30th, paracentesis was performed, which the lady bore with great fortitude and good spirits. During the latter months of pregnancy she became extremely heavy and unwieldy, especially before the two last tappings. She was safely delivered of a strong full-grown boy on the 24th January, 1792, the labour having been quick and easy. In eleven days after delivery paracentesis was again performed. After this, until a sudden change which caused her death, her general health appeared rather to improve. The cause of death was a collection of matter in one of the ovaria, which burst. Sir L. observes that this is the first case which he has met with on record, where the mother and child survived the delivery. He will see that this is an oversight, as Mr. Laurie's case, quoted above, will prove.

Upon the whole, the notice which has been taken of Mr. Langstaff's paper has elicited, from various quarters, much useful and interesting information on this subject, which cannot but prove welcome to the practitioner both of medicine and surgery.

---

### III.
### RETROSPECTIONS.

1. *Divided Arteries.*—The principal object of Dr. Jones's experiments was to prove that a plug was formed at the extremity of the vessel, by which the bleeding was arrested—and that the effect of a ligature was to produce accretion of the sides of the vessel by dividing their inner coats. Petit and Morand demonstrated, almost exactly, the same things, nearly one hundred years ago, in the *Mem. de l'Acad. des Sciences*, for 1731-2-3-4-5. See also *Medical Observations and Enquiries, vol.* iv. *p.* 484, *and vol.* v. *p.* 955.

2. *Fowler's Solution.*—It is curious that the erudite Dr. Parr, and the no less erudite Dr. Paris should have given Dr. Fowler the credit of having first introduced the arsenical solution in its present form, as a substitute for Edwards's tasteless ague drop. Yet, long before either of these was talked of, the present identical preparation was described and given in agues. We have no hesitation in asserting that Dr. Fowler copied the prescription, and published it as his own. But now for proof.

In the first volume of the Medical Museum, page 433, the reader will see the following passage.

"The celebrated John Christ. Jacobo (see *Act. Acad. Elect. Moguntl. secent. util.* which is at Erfurth, in Thuringia, tom. 1, p. 216) describes an arsenical febrifuge, prepared in this manner.

℞ Arsen. Albi, p. j.
Sal. Tart. p. xij.
Aquæ, p. 168.

"This mixture is boiled until the water is consumed to one half. After it cools, the evaporated part of the water is again added, together with a little spirit of wine for its better conservation.

"He recommends this remedy as good for fevers of various kinds. It is given to adults to 30 drops, but to infants are given six, eight, or ten drops."

The Article in the Medical Museum was printed in 1758, and the writer mentions his stopping agues with the medicine.

The preparation, as every one may see, is the identical Fowler's solution, except that it is rather weaker; *viz.* 1 part of arsenic to 168 of the menstruum, while Dr. Fowler's form is 1 part of arsenic to 120 of the solution. We again repeat the charge of copyism against Dr. Fowler.

"*Palmam qui meruit ferat.*"

IV.

*The COMMITTEE appointed to carry into effect the plan for establishing a National Benevolent Medical Fund, submit, to the consideration of the Profession, the following Prospectus of a Company, to be entitled*

### THE SURGEON AND APOTHECARIES' DRUG COMPANY,

### AND BENEVOLENT MEDICAL INSTITUTION.

The advantages contemplated by the Committee, in the success of this undertaking, are fourfold:—1stly, In the supply of *genuine* and *unmixed* Drugs and Chemicals; an object *utterly unattainable* under the present system.—2ndly, In a saving to the buyer of 20 per cent. on the prices hitherto established.—3rdly, In affording a certain and competent resource for the incapacitated practitioner, who has been unable to provide against the unfortunate contingencies of life.—And 4thly, In securing to widows and orphans such a provision as shall place them above penury and distress, should every other resource be wanting. To effect these desirable objects, the Committee submit the following propositions:—

1st, To raise by shares of £25. each, a capital of £100,000. Subscribers to be practising Surgeons and Apothecaries; and shares to be transferable to practitioners only. An advance of 20 per cent. to be paid at the time of subscribing, and instalments of 20 per cent. to be called for as required; a guarantee being, however, given, that the whole of such instalments shall not be demanded within twelve months from the date of subscription.

2ndly, That no Subscriber be entitled, either for himself or his family, to the benefit of the Benevolent Fund, who holds less than *two* shares.

3rdly, That a dividend of 5 per cent. per annum on the capital advanced, be paid half yearly to each Subscriber, and that the first profits of the Establishment be added to the Company's capital, until every original share of £25. amounts in value to the sum of £37. 10*s*. which will give to each Subscriber an annual interest of 7½ per cent. on his capital actually advanced.

4thly, That as soon as the above increase in capital shall be effected (which the Committee calculate will be done in two years), all future profits to be appropriated to the Benevolent Fund, but no payment to be made to any claimant within three years of this time, unless the amount of the Benevolent Fund exceed the sum of £50,000.

5thly, That the *Benevolent Institution* consist of a Patron and Vice-Patrons, who, with the Directors of the Company for the time being, shall hold in trust the funds appropriated for this purpose.

6thly, To allow an annuity of £100. for life to every Subscriber who retires from business under *ill-health or infirmity*, with a less income than £150. per year.

7thly, To allow to every widow (of Subscribers) the annual sum of £60. during her widowhood, provided she does not possess, from any other resource, an income exceeding the stipend from the Benevolent Fund; and to each child the annual sum of £10. until they attain the age of twenty-one years.

8thly, To grant to the widows and children of such Subscribers as have retired upon the benefits of the Benevolent Fund, the same provisions as the above.

9thly, That the value of the shares of every Subscriber, who retires or dies, be repaid to his representatives or assigns, by the Company, unless he shall have participated, or his widow and children claim to participate, in the benefits of the Benevolent Fund; in either of which case, the shares of such Subscriber shall revert to the Company without fee or purchase.

10thly, In the case of transfer of shares, no benefit to accrue, either to the purchaser or his family, until five years from the date of such transfer.

11thly, That such professional gentlemen, who do not practise pharmacy, be invited to support the Institution, by paying an annual sum of *two guineas*, which will entitle them and their families (if needful) to the benefit of the Benevolent Fund.

Communications to be addressed to the Committee, at Mr. J. CHURCHILL'S, Bookseller, *Saville House, Leicester Square*, where Books are opened to receive the names of Subscribers. *Letters will not be received except post free.*

### V.
### EXPLANATION OF MR. WEISS'S PLATE.

A, a small handle which turns the borer.
B, the handle of the instrument.
C, a slide for moving the borer forward or backwards, as requires.
D, a brass wheel, to which a bow may be attached to promote the action of the borer.
E. an upright for opening or shutting the instrument, by drawing it backwards, or pushing it forwards with the thumb.
F, a catch, to strengthen the hold of the instrument, and to prevent E from slipping backward.
G, is the borer shewn between the blades of the open instrument.
H, the blades closed. H. H. the blades expanded, shewing the borer.
I, a screw for tightening the string of the bow, when too loose.
K, the bow itself.
L, an instrument that was made with 3 blades, but not considered so good as the one with two; for it increases the difficulty to catch the stone.

To open the instrument, the thumb must be pressed upon F and E, drawn towards D, which is shewn underneath. To close the instrument, E must be pushed forward. When the instrument is in the bladder, and opened, the borer must be withdrawn by bringing C towards A, and when the instrument has grasped the stone, C is again moved forward, so as to bring the borer, G, in contact with the stone, and perforate it, by turning A, and pressing C gently forward.

The bow is used by putting the string twice round the wheel, and then letting it in the slit at the end of the bow, by moving which backwards or forwards, the borer is turned much quicker than with the small handle, A.

I have the pleasure to inform the public, that I am now taking out a patent for an Enema Syringe, which will surpass any thing of the kind hitherto produced for that purpose, or for relieving the stomach of poison. I shall take the first opportunity of laying it before the public.

### VI.
### ELEGANT WARM, COLD, VAPOUR BATHS, &c.

Mr. SEAMAN has fitted up, in a very superior manner, every kind of warm, vapour, cold, and shampooing baths, at his house, *No. 9, Suffolk Place, Haymarket,* where the Faculty and the Public will meet with every accommodation, and on the most moderate terms.

9, *Suffolk Place, 22d Dec.* 1824.

### XIV.
### TO CORRESPONDENTS.

The Medical Practice advertised in our last Number has been long since disposed of, and right happy we are to have an opportunity of making this announcement, as it has cost us more than *five pounds* in postage already. The thoughtless practice of directing letters, on such occasions, without paying the postage, is reprehensible; but we are forced to expose the conduct of

### MR. BATTLEY ON OPIUM.

*To the Editor of the Medico-Chirurgical Review.*

SIR,

In submitting to your attention the annexed list, I beg leave to remark, particularly, upon the properties of the Liquor Opii Sedativus. This medicine possesses the narcotic and sedative, without the exciting property of Opium. It diminishes the action of the heart and arteries, lessens the sensibility of the nervous system, and allays irritation, pain, and cough, even in diseases in which the inflammatory diathesis prevails.

I am induced to solicit your attention more especially to this preparation, not only in consideration of its great and acknowledged importance in the treatment of disease, but from the circumstance of the medicine having been impaired in its reputation by the introduction of a preparation under the same name. This preparation, upon analysis, I find to be;

Diluted ligneous acid, holding in solution morphium.

Colouring matter, which upon being precipitated, is again soluble in dilute liq. potassæ, but not in water or alcohol.

A gum resin which assimulates to matter precipitated from tincture of henbane, and a small portion of a resin of a different description, but which I cannot particularly define.

Comparing this mixture with the Liquor Opii Sedativus, which I prepare, it may be proper to add, that the latter does not contain *morphium*, or if any, a very trifling portion only, and applying these preparations, severally, to the animal economy, I am of opinion that the first, by the resinous and other matter combined with morphium, would produce nervous irritation: and experience shows that the second *allays that affection* to an extent entitling it to a high character in *diseases of irritation.*

I am, SIR,

Your obedient Servant,

RICHARD BATTLEY.

FORE STREET, CRIPPLEGATE, LONDON.

DECEMBER 20th, 1824.

'UM Colchici

Scillæ

UM Aceticum Per Retort

.. Fortissimus

Benzoicum

Citricum

Hydrocyanicum

Muriaticum

Nitricum

Nitrosum

Sulphuricum S. G.

Tartaricum

:R Sulphuricus S. G.

Rectificatus S. G.

IOL S. G.

Spicatæ Extractum

EN Exsiccatum

NIÆ Murias

Subcarbonas

ONIUM Tartarizatum

ONII Sulphuretum Præcipit

Cassiæ

Cinnamomi

Rosæ

TI Nitras

Capsici

IUM Copaibæ

Styracis

HI Subnitras

IA

RA

ARIS

EUM

U

M Sabinæ

Saponis

*ine*

NTHIDIS Pulpa

TIO Amygdalarum

Aromatica

Aurantiorum

Opii

Piperis Nigri

CONFECTIO Rosæ Caninæ

.. .. Gallicæ

.. Sennæ

CORTEX Aurantii

.. Canellæ

.. Cascarillæ

.. Cinchonæ Cordifoliæ

.. .. Lancifoliæ

.. .. Oblongifoliæ

.. Cinnamomi

.. Cuspariæ

.. Limonum

.. Mezerei

.. Rad. Sarsaparillæ

COCCUS

CROCI Stigmata

CUBEBA

*Emetine*

EMPLASTRUM Ammoniaci

.. .. cum Hydrargyro

.. Belladonnæ

.. Conii

.. Cumini

.. Galbani Compositum

.. Hydrargyri

.. Cantharidis

.. Opii

.. .. Sedativum

.. Picis Compositum

.. Plumbi

.. Resinæ

.. Saponis

.. Stramonii

.. Thuris Compositum

EXTRACTUM Aconiti

.. Aloes Purificatum

.. Anthemidis

.. Belladonnæ

.. Capsulis Stramonii

.. Cascarillæ

.. Cinchonæ

.. .. Resinosum

.. Colchici Aquosum

.. .. Acetosum

EXTRACTUM Colchici [illegible]

.. Colocynth[illegible]

.. ..

.. Conii

.. Elaterii

.. Gentianæ

.. Gratiolæ

.. Hæmatox[illegible]

.. Hellebori [illegible]

. Humuli

.. Hyoscyami

.. Jalapæ

.. .. Res[illegible]

.. Lactucæ

.. .. Vi[illegible]

.. Mezerei C[illegible]

.. Nucis Vom[illegible]

.. ..

.. Opii

.. Papaveris

. Rhei

.. Sarsaparillæ

.. ..

.. Semina Str[illegible]

.. Tabaci

.. Taraxaci

.. Uvæ Ursi

FERRI Ammoniatum

.. Subcarbonas

.. Sulphas

.. Tartarizatum

FOLIA Belladonnæ

.. Conii

.. Digitalis

.. Hyoscyami

.. Sennæ

.. Stramonii

.. Uvæ Ursi

GUMMI Acaciæ

.. Ammoniaci

.. Assafœtidæ

.. Benzoini

.. Galbani

.. Guaiaci

Oleum Cinnamomi

.. Juniperi

.. Lavandulæ

.. Menthæ Piperitæ

.. .. Viridis

.. Myristicæ

.. Pimentæ

.. Pulegii

.. Ricini, E. I.

.. .. W. I.

.. Rosmarini

.. Sabinæ

.. Terebinthinæ

.. .. Rectificatum

Opium

Opii Extractum Sedativum

.. .. Resinosum

.. Liquor Sedativus

Oxymel Scillæ

Pilulæ Aloes cum Myrrha

.. Ferri Compositæ

.. Galbani ..

.. Hydrargyri

.. .. Submur. Comp.

.. Saponis cum Opio

.. Scillæ Compositæ

Plumbi Acetas

Potassæ Acetas

.. Carbonas

.. Subcarbonas

.. Sulphas

.. Sulphuretum

.. Supertartras

.. Tartras

Pulvis Aloes Compositus

.. Antimonialis

.. Arsenici

.. Belladonnæ

.. Calumbæ

.. Cascarillæ

.. Cinchonæ Cordifolæ

.. .. Lancifoliæ

.. .. Oblongifoliæ

.. Cinnamomi

Pulvis Cinnamomi Compositu

.. Conii

.. Colchici

.. Corticis Sarsaparillæ

.. Cretæ Compositus

.. .. .. cu

.. Cubebæ

.. Digitalis

.. Guaiaci

.. Hyoscyami

.. Ipecacuanhæ

.. .. Composi

.. Jalapæ

.. Myrrhæ

.. Opii

.. Rhei

.. Sabinæ

.. Sarsaparillæ

.. Scammoneæ

.. .. Compositu

.. Scammoneæ cu Calon

.. Scillæ

.. Sennæ

.. Tragacanthæ

.. .. Composi

.. Uvæ Ursi

.. Zingiberis

*Quinina*

.. *Sulphas*

Radix Columbæ

.. Colchici Siccatus

.. Gentianæ

.. Glycyrrhizæ

.. Hellebori

.. .. Nigri

.. Ipecacuanhæ

.. Jalapæ

.. Mezerei

.. Rhei

.. Sarsaparillæ

.. Scillæ Siccatus

.. Serpentariæ

.. Zingiberis

### Extra Limites.

Cardamomi

[illegible] Colchici

Stramonii

[illegible]artarizata

[illegible]arbonas

[illegible]ubcarbonas

[illegible]s Ætheris Aromat. S. G.

.. Nitrici, S. G.

.. Sulphurici, S. G.

.. Comp. S. G.

Ammoniæ, S. G.

.. Aromaticus

.. Colchici

.. Fœtidas

.. Succinatus

Armoraciæ Compositus

Juniperi Compositus

Lavandulæ

.. Compositus

Myristicæ

